# 1-Cyano­methyl-1,4-diazo­niabicyclo­[2.2.2]octane tetra­bromidocadmate(II)

**DOI:** 10.1107/S1600536810047495

**Published:** 2010-11-27

**Authors:** Bin Wei

**Affiliations:** a Ordered Matter Science Research Center, Southeast University, Nanjing 211189, People’s Republic of China

## Abstract

In the title salt, (C_8_H_15_N_3_)[CdBr_4_], four Br atoms coordinate the Cd^II^ atom in a distorted tetra­hedral geometry. In the crystal, weak N—H⋯Br inter­actions connect the anion to three symmetry-related cations. The crystal structure also displays very weak C—H⋯Br inter­actions.

## Related literature

For background to 1,4-diaza­bicyclo­[2.2.2]octane derivatives and their properties, see: Basavaiah *et al.* (2003 ▶); Chen *et al.* (2010 ▶); Wang *et al.* (2005 ▶); Xiong *et al.* (2002 ▶); Ye *et al.* (2006 ▶).
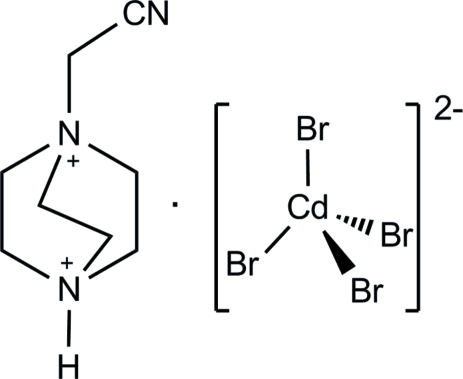


## Experimental

### 

#### Crystal data

(C_8_H_15_N_3_)[CdBr_4_]
*M* *_r_* = 585.27
Monoclinic, 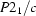

*a* = 8.610 (3) Å
*b* = 14.071 (4) Å
*c* = 12.702 (4) Å
β = 94.136 (4)°
*V* = 1534.9 (8) Å^3^
*Z* = 4
Mo *K*α radiation
μ = 11.82 mm^−1^
*T* = 293 K
0.2 × 0.2 × 0.2 mm

#### Data collection

Rigaku Mercury CCD diffractometer
Absorption correction: multi-scan (*CrystalClear*; Rigaku, 2005 ▶) *T*_min_ = 0.470, *T*_max_ = 1.000
16630 measured reflections
3518 independent reflections
2861 reflections with *I* > 2σ(*I*)
*R*_int_ = 0.068

#### Refinement

*R*[*F* ^2^ > 2σ(*F* ^2^)] = 0.033
*wR*(*F* ^2^) = 0.079
*S* = 0.76
3518 reflections
145 parameters
H-atom parameters constrained
Δρ_max_ = 0.61 e Å^−3^
Δρ_min_ = −0.88 e Å^−3^

### 

Data collection: *CrystalClear* (Rigaku, 2005 ▶); cell refinement: *CrystalClear*; data reduction: *CrystalClear*; program(s) used to solve structure: *SHELXS97* (Sheldrick, 2008 ▶); program(s) used to refine structure: *SHELXL97* (Sheldrick, 2008 ▶); molecular graphics: *SHELXTL* (Sheldrick, 2008 ▶); software used to prepare material for publication: *SHELXL97*.

## Supplementary Material

Crystal structure: contains datablocks I, global. DOI: 10.1107/S1600536810047495/bh2319sup1.cif

Structure factors: contains datablocks I. DOI: 10.1107/S1600536810047495/bh2319Isup2.hkl

Additional supplementary materials:  crystallographic information; 3D view; checkCIF report

## Figures and Tables

**Table 1:** Hydrogen-bond geometry (Å, °)

*D*—H⋯*A*	*D*—H	H⋯*A*	*D*⋯*A*	*D*—H⋯*A*
N1—H1*C*⋯Br1^i^	0.90	2.85	3.466 (4)	127
N1—H1*C*⋯Br2^ii^	0.90	2.69	3.325 (4)	128
N1—H1*C*⋯Br4^i^	0.90	3.11	3.711 (4)	126
C2—H2*B*⋯Br3^iii^	0.97	2.83	3.765 (5)	162
C4—H4*B*⋯Br1	0.97	2.85	3.643 (4)	140
C7—H7*A*⋯Br3^iv^	0.97	2.90	3.626 (4)	132
C7—H7*B*⋯Br2^iii^	0.97	2.78	3.683 (4)	154

Symmetry codes: (i) 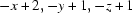; (ii) 
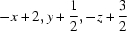
; (iii) 
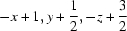
; (iv) 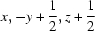.

**Fig. 1.**
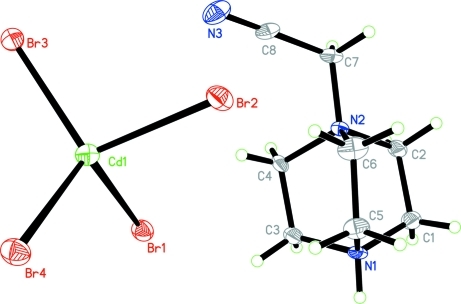
The structure of the title compound with labeling scheme and displacement ellipsoids drawn at the 30% probability level.

**Fig. 2.**
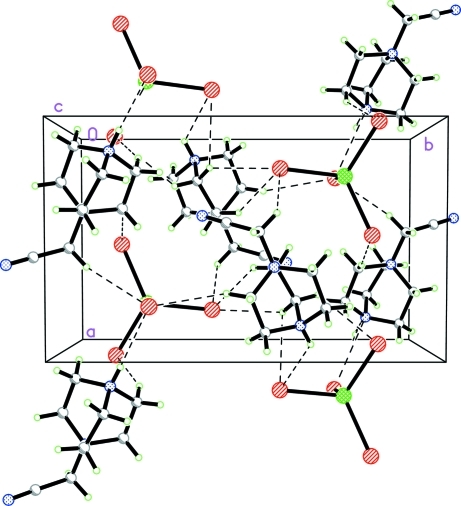
The crystal structure of the title compound viewed down the c axis. Intermolecular interactions are shown as dashed lines.

## supplementary crystallographic information

### Comment

We are studying the dielectric-ferroelectric materials. 1,4-Diazabicyclo[2.2.2]octane (DABCO) has attracted attention in recent years because of its nucleophilicity (Basavaiah *et al.*, 2003; Xiong *et al.*, 2002) and ferroelectric properties of its derivatives (Chen *et al.*, 2010). For a project on the electric properties of DABCO derivatives (Ye *et al.*, 2006), the title compound was prepared. With no dielectric anomaly observed, the title compound should not be a real ferroelectrics or there may be no distinct phase transition occurring within the measured temperature range (Wang *et al.*, 2005).

The asymmetric unit of the title compound is shown in Fig 1. The Cd atoms are coordinated by four Br atoms with very similar distances in the range 2.5764 (10) to 2.6195 (12) Å. The Br—Cd—Br bond angles are between 98.29 (3) and 116.85 (4)°, which show that the coordination polyhedron can be described as an irregular tetrahedron. Cations (C_8_H_14_N_3_)^2+^ and anions CdBr_4_^2-^ are connected *via* weak hydrogen bonds. Weak C—H···Br intramolecular and intermolecular hydrogen bonds also contribute to the stability of the crystal structure, forming one-dimensional chains running along the *a* axis (Fig. 2).

### Experimental

1,4-Diaza-bicyclo[2.2.2]octane (DABCO) (10 mmol, 1.14 g) and bromoacetonitrile (20 mmol, 2.4 g) were dissolved in CH_3_CN (10 ml) under stirring for 1 h. at room temperature. 1-(Cyanomethyl)-4-aza-1-azonia-bicyclo[2.2.2]octane bromide was obtained by filtering the solid precipitate, then washed with acetonitrile and dried (yield: 90%).

CdBr_2_ (10 mmol, 0.271 g) and 4 ml 60% HBr were dissolved in MeOH (20 ml) and 1-(cyanomethyl)-4-aza-1-azonia-bicyclo[2.2.2]octane bromide (20 mmol, 0.464 g) dissolved in 10 ml of methanol was added. The mixture was stirred until the solution was clear. After slow evaporation (5 days) of the solvent, colourless plate crystals of the title compound were obtained in about 56% yield.

### Refinement

H atoms bonded to C and N atoms were placed in idealized positions [C—H = 0.97 Å and N—H = 0.90 Å] and allowed to ride on their parent atoms with *U*_iso_ fixed at 1.2 *U*_eq_(Carrier atom).

### Crystal data

**Table:**

(C_8_H_15_N_3_)[CdBr_4_]	*F*(000) = 1088
*M**_r_* = 585.27	*D*_x_ = 2.533 Mg m^−^^3^
Monoclinic, *P*2_1_/*c*	Mo *K*α radiation, λ = 0.71073 Å
Hall symbol: -P 2ybc	Cell parameters from 4263 reflections
*a* = 8.610 (3) Å	θ = 2.4–27.5°
*b* = 14.071 (4) Å	µ = 11.82 mm^−^^1^
*c* = 12.702 (4) Å	*T* = 293 K
β = 94.136 (4)°	Prism, colourless
*V* = 1534.9 (8) Å^3^	0.2 × 0.2 × 0.2 mm
*Z* = 4	

### Data collection

**Table:**

Rigaku Mercury CCD diffractometer	3518 independent reflections
Radiation source: fine-focus sealed tube	2861 reflections with *I* > 2σ(*I*)
graphite	*R*_int_ = 0.068
Detector resolution: 28.5714 pixels mm^-1^	θ_max_ = 27.5°, θ_min_ = 3.1°
ω scans	*h* = −11→11
Absorption correction: multi-scan (*CrystalClear*; Rigaku, 2005)	*k* = −18→18
*T*_min_ = 0.470, *T*_max_ = 1.000	*l* = −16→16
16630 measured reflections	

### Refinement

**Table:**

Refinement on *F*^2^	Primary atom site location: structure-invariant direct methods
Least-squares matrix: full	Secondary atom site location: difference Fourier map
*R*[*F*^2^ > 2σ(*F*^2^)] = 0.033	Hydrogen site location: inferred from neighbouring sites
*wR*(*F*^2^) = 0.079	H-atom parameters constrained
*S* = 0.76	*w* = 1/[σ^2^(*F*_o_^2^) + (0.0502*P*)^2^ + 0.2386*P*] where *P* = (*F*_o_^2^ + 2*F*_c_^2^)/3
3518 reflections	(Δ/σ)_max_ = 0.018
145 parameters	Δρ_max_ = 0.61 e Å^−^^3^
0 restraints	Δρ_min_ = −0.88 e Å^−^^3^
0 constraints	

### Fractional atomic coordinates and isotropic or equivalent isotropic displacement parameters (Å^2^)

**Table:**

	*x*	*y*	*z*	*U*_iso_*/*U*_eq_	
Cd1	0.78773 (4)	0.22670 (2)	0.50861 (3)	0.02924 (11)	
Br1	0.82000 (6)	0.40556 (3)	0.46315 (4)	0.03047 (13)	
Br2	0.79028 (6)	0.24284 (3)	0.71414 (4)	0.03420 (13)	
Br3	0.52068 (6)	0.15357 (3)	0.45098 (4)	0.03464 (13)	
Br4	1.03019 (6)	0.13565 (3)	0.45657 (4)	0.03694 (14)	
C8	0.4254 (6)	0.4480 (3)	0.6975 (4)	0.0314 (11)	
N2	0.6179 (4)	0.5753 (2)	0.7314 (3)	0.0205 (7)	
C5	0.9032 (5)	0.5513 (3)	0.7405 (4)	0.0328 (11)	
H5A	0.9784	0.5564	0.8008	0.039*	
H5B	0.9444	0.5081	0.6898	0.039*	
N1	0.8754 (4)	0.6469 (2)	0.6913 (3)	0.0242 (8)	
H1C	0.9610	0.6750	0.6691	0.029*	
C6	0.7506 (6)	0.5138 (3)	0.7750 (4)	0.0378 (12)	
H6A	0.7350	0.4490	0.7501	0.045*	
H6B	0.7532	0.5132	0.8515	0.045*	
C4	0.6267 (5)	0.5849 (3)	0.6140 (3)	0.0294 (10)	
H4A	0.5365	0.6195	0.5842	0.035*	
H4B	0.6263	0.5224	0.5819	0.035*	
C3	0.7744 (5)	0.6376 (3)	0.5904 (3)	0.0283 (10)	
H3A	0.8293	0.6027	0.5387	0.034*	
H3B	0.7486	0.7000	0.5620	0.034*	
C2	0.6364 (6)	0.6733 (3)	0.7808 (4)	0.0316 (11)	
H2A	0.5586	0.7160	0.7484	0.038*	
H2B	0.6215	0.6696	0.8557	0.038*	
C7	0.4643 (5)	0.5354 (3)	0.7579 (3)	0.0279 (10)	
H7A	0.4677	0.5215	0.8328	0.034*	
H7B	0.3835	0.5825	0.7424	0.034*	
N3	0.3933 (6)	0.3830 (3)	0.6497 (4)	0.0459 (12)	
C1	0.7981 (5)	0.7107 (3)	0.7646 (4)	0.0300 (10)	
H1A	0.7909	0.7744	0.7354	0.036*	
H1B	0.8590	0.7135	0.8318	0.036*	

### Atomic displacement parameters (Å^2^)

**Table:**

	*U*^11^	*U*^22^	*U*^33^	*U*^12^	*U*^13^	*U*^23^
Cd1	0.02631 (19)	0.03071 (19)	0.0309 (2)	−0.00124 (14)	0.00314 (15)	0.00137 (14)
Br1	0.0324 (3)	0.0264 (2)	0.0335 (3)	0.00131 (19)	0.0088 (2)	−0.00169 (18)
Br2	0.0285 (3)	0.0477 (3)	0.0266 (3)	0.0031 (2)	0.0034 (2)	0.0044 (2)
Br3	0.0287 (3)	0.0421 (3)	0.0334 (3)	−0.0064 (2)	0.0042 (2)	−0.0070 (2)
Br4	0.0334 (3)	0.0351 (3)	0.0433 (3)	0.0054 (2)	0.0086 (2)	0.0016 (2)
C8	0.029 (3)	0.026 (2)	0.039 (3)	−0.008 (2)	0.003 (2)	0.010 (2)
N2	0.0180 (18)	0.0228 (18)	0.0213 (18)	0.0003 (14)	0.0053 (15)	0.0002 (14)
C5	0.023 (2)	0.032 (3)	0.043 (3)	0.007 (2)	0.003 (2)	0.002 (2)
N1	0.0148 (18)	0.031 (2)	0.029 (2)	−0.0028 (15)	0.0094 (15)	−0.0017 (15)
C6	0.027 (3)	0.034 (3)	0.052 (3)	0.008 (2)	−0.003 (2)	0.015 (2)
C4	0.027 (3)	0.045 (3)	0.016 (2)	−0.004 (2)	0.0037 (19)	−0.0024 (18)
C3	0.025 (2)	0.035 (3)	0.026 (2)	−0.002 (2)	0.007 (2)	0.0022 (18)
C2	0.033 (3)	0.031 (2)	0.033 (3)	−0.003 (2)	0.015 (2)	−0.013 (2)
C7	0.020 (2)	0.033 (2)	0.032 (3)	−0.0025 (19)	0.0067 (19)	0.0061 (19)
N3	0.051 (3)	0.032 (2)	0.053 (3)	−0.012 (2)	−0.004 (2)	0.011 (2)
C1	0.028 (2)	0.028 (2)	0.035 (3)	−0.003 (2)	0.008 (2)	−0.0084 (19)

### Geometric parameters (Å, °)

**Table:**

Cd1—Br3	2.5766 (8)	N1—H1C	0.8997
Cd1—Br4	2.5760 (8)	C6—H6A	0.9700
Cd1—Br1	2.6015 (9)	C6—H6B	0.9700
Cd1—Br2	2.6191 (10)	C4—C3	1.521 (6)
C8—N3	1.122 (6)	C4—H4A	0.9700
C8—C7	1.475 (6)	C4—H4B	0.9700
N2—C7	1.497 (5)	C3—H3A	0.9700
N2—C6	1.506 (5)	C3—H3B	0.9700
N2—C4	1.505 (5)	C2—C1	1.516 (6)
N2—C2	1.519 (5)	C2—H2A	0.9700
C5—N1	1.495 (5)	C2—H2B	0.9700
C5—C6	1.510 (7)	C7—H7A	0.9700
C5—H5A	0.9700	C7—H7B	0.9700
C5—H5B	0.9700	C1—H1A	0.9700
N1—C1	1.485 (5)	C1—H1B	0.9700
N1—C3	1.501 (5)		
			
Br3—Cd1—Br4	116.84 (3)	N2—C4—C3	110.0 (3)
Br3—Cd1—Br1	115.51 (2)	N2—C4—H4A	109.7
Br4—Cd1—Br1	108.83 (2)	C3—C4—H4A	109.7
Br3—Cd1—Br2	105.13 (2)	N2—C4—H4B	109.7
Br4—Cd1—Br2	110.50 (2)	C3—C4—H4B	109.7
Br1—Cd1—Br2	98.28 (2)	H4A—C4—H4B	108.2
N3—C8—C7	178.1 (5)	N1—C3—C4	108.4 (3)
C7—N2—C6	111.2 (3)	N1—C3—H3A	110.0
C7—N2—C4	111.4 (3)	C4—C3—H3A	110.0
C6—N2—C4	109.0 (3)	N1—C3—H3B	110.0
C7—N2—C2	108.3 (3)	C4—C3—H3B	110.0
C6—N2—C2	108.4 (4)	H3A—C3—H3B	108.4
C4—N2—C2	108.5 (3)	C1—C2—N2	109.2 (3)
N1—C5—C6	108.6 (4)	C1—C2—H2A	109.8
N1—C5—H5A	110.0	N2—C2—H2A	109.8
C6—C5—H5A	110.0	C1—C2—H2B	109.8
N1—C5—H5B	110.0	N2—C2—H2B	109.8
C6—C5—H5B	110.0	H2A—C2—H2B	108.3
H5A—C5—H5B	108.3	C8—C7—N2	111.5 (4)
C5—N1—C1	110.3 (3)	C8—C7—H7A	109.3
C5—N1—C3	110.1 (3)	N2—C7—H7A	109.3
C1—N1—C3	109.2 (3)	C8—C7—H7B	109.3
C5—N1—H1C	114.6	N2—C7—H7B	109.3
C1—N1—H1C	110.2	H7A—C7—H7B	108.0
C3—N1—H1C	102.0	N1—C1—C2	109.4 (3)
N2—C6—C5	110.1 (4)	N1—C1—H1A	109.8
N2—C6—H6A	109.6	C2—C1—H1A	109.8
C5—C6—H6A	109.6	N1—C1—H1B	109.8
N2—C6—H6B	109.6	C2—C1—H1B	109.8
C5—C6—H6B	109.6	H1A—C1—H1B	108.2
H6A—C6—H6B	108.1		

### Hydrogen-bond geometry (Å, °)

**Table:**

*D*—H···*A*	*D*—H	H···*A*	*D*···*A*	*D*—H···*A*
N1—H1C···Br1^i^	0.90	2.85	3.466 (4)	127
N1—H1C···Br2^ii^	0.90	2.69	3.325 (4)	128
N1—H1C···Br4^i^	0.90	3.11	3.711 (4)	126
C2—H2B···Br3^iii^	0.97	2.83	3.765 (5)	162
C4—H4B···Br1	0.97	2.85	3.643 (4)	140
C7—H7A···Br3^iv^	0.97	2.90	3.626 (4)	132
C7—H7B···Br2^iii^	0.97	2.78	3.683 (4)	154

Symmetry codes: (i) −*x*+2, −*y*+1, −*z*+1; (ii) −*x*+2, *y*+1/2, −*z*+3/2; (iii) −*x*+1, *y*+1/2, −*z*+3/2; (iv) *x*, −*y*+1/2, *z*+1/2.
